# Teachers’ Professional Development and Intelligent Ways of Coping with It: A Systematic Review in Elementary and Middle School Education

**DOI:** 10.3390/jintelligence11010001

**Published:** 2022-12-21

**Authors:** Juan Antonio Salmerón Aroca, Pedro Moreno Abellán, Silvia Martínez de Miguel López

**Affiliations:** Department of Theory and History of Education, Faculty of Education, University of Murcia, 30100 Murcia, Spain

**Keywords:** teachers, professional development, smart schools, training, competences, generational diversity

## Abstract

This research addresses teacher training at different generational stages, with the aim of analysing the training actions developed by school teachers and the intentionality of linking them to their professional development, in order to offer a broad perspective of the paradigm of smart schools, allowing for the adjustment of the quality of training to real demands. To this end, a systematic review of articles published between 2012 and 2022 in the main databases (WoS, Scopus, Eric, Dialnet, and Google Scholar) was carried out. After applying the inclusion criteria, 56 articles were selected and analysed following the PRISMA 2020 statement. The findings showed the interest and importance of initial, continuous, and lifelong learning among teachers as a driver of professional development. The results also showed that research is mostly focused on novice teachers and qualitative methodologies predominate, although this is limited to certain countries and specialised publications. However, generational differences were observed. While younger teachers are more highly trained in ICT, older teachers have a higher level of competence at a processual and relational level in the classroom. In conclusion, it should be noted that teacher training linked to professional development has an impact on school improvement, especially if it is carried out from an intergenerational collaborative perspective, and the acquisition of new skills.

## 1. Introduction and State of Play

The professional development of school teachers is a continuous process of improving teachers’ skills and knowledge (Richter et al. 2011), and intelligent ways of dealing with it involve approaching this process effectively and efficiently. This can include participation in training and development programs, the development of pedagogical and teaching skills, and the implementation of innovative and effective approaches in the classroom (Preston et al. 2017).

We are currently facing a knowledge society exposed to the effects of globalisation that impact economic, political, and social models, without rethinking the reality of certain contexts of formal or regulated education, which can be better adapted to society’s characteristics or needs. These are also important issues that directly affect the loss of values, the integration of equality rights, environmental awareness, and the basis of emotional intelligence, as well as the dizzying scientific–technological progress that, in turn, favours the speculation of knowledge. According to Mota (2020), the educational community must manifest an integral vision of those strategies that define intelligent and efficient decision making throughout the learning process in order to adapt to new challenges.

In pursuit of these objectives, authors such as Pamies et al. (2022) have stated that teachers’ professional development plans are the primary mechanisms of action to improve the services offered by educational institutions. This is why professional development issues occupy a prominent place in European public policies, motivated, among other reasons, by the concerns of education professionals themselves regarding their professional development. From this point of view, it could be said that all countries are investigating how to improve their schools in order to respond more adequately to the social and economic needs of their environment. In this context, the most significant resource in schools is the teaching staff.

The Eurydice report (European Commission 2018) on the teaching profession in Europe, which develops access, promotion, and external support for teachers, provides a comparative picture of national policies on teaching careers in Europe. It points out that higher-education institutions are responsible for planning the number of teachers needed on an annual basis, which is based on the ratio of current teachers rather than on future needs. This planning is therefore hampered by two main factors, which are common denominators: The shortage of teachers and the ageing of the teaching staff. Furthermore, in the Teaching and Learning International Survey (TALIS) conducted by the Organisation for Economic Co-operation and Development (Jerrim and Sims 2019), those aspects that refer to professional development are highlighted. In the case of in-service training, it is corroborated by the fact that the majority of teachers, both primary and secondary, completed teacher development courses in the last year among teachers in England.

**Figure 1:**
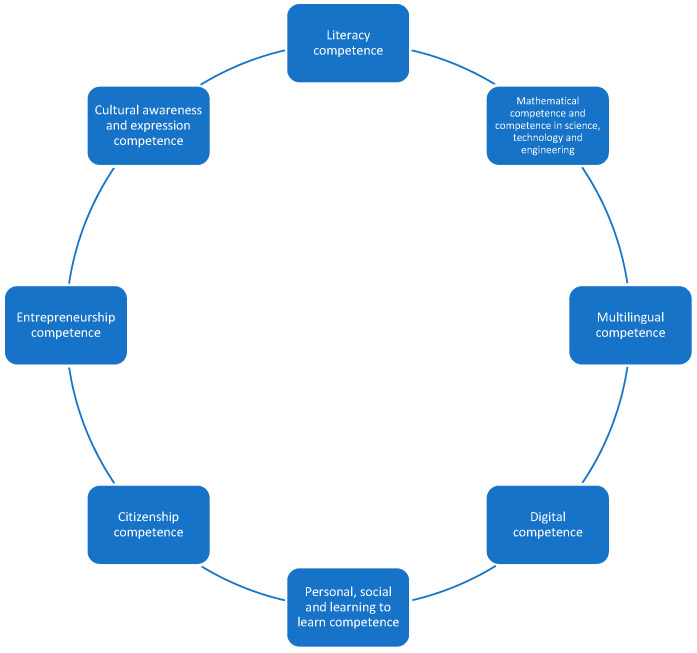
Professional competence model. Source: European Commission/EACEA/Eurydice (2018).

With all of this, it is vitally important at the competence level to consider continuous teacher training as a driving, integrating, and transforming element that motivates participation. In the same way, it is also important to determine whether this training contemplates the processes of teacher collaboration in its different modalities (Krischesky and Murillo 2018) and between teachers of different generations, taking into account age as a relevant factor in the shaping of educational institutions (Portela et al. 2020). This is also the case for reflections on the change in teachers’ mentality by granting new models of action. We are focused, of course, on new competences of a procedural nature that guarantee a change in paradigm within the teaching–learning process of any educational community for the transformation of knowledge. In this context, and from a more concrete and applied perspective, as recognised by González and Escudero (2017), this path of action must be followed in classroom programmes, in order to understand, among other aspects, the social reality in which students live and act. Even knowing that the treatment of technical–scientific learning is gaining ground over the socio-critical nature (taking into account the handicap of transversality), the current curriculum tends to monopolise theoretical content on instrumental subjects excessively, which does not allow the pragmatism of spaces for training to re-emerge. It seems clear from the above that it is not possible to improve the educational action of teachers and, therefore, their professional development without improving the functioning of schools in parallel (Vélaz de Medrano and Vaillant 2009). It is in this conceptual framework that the need to accommodate the assumptions of the smart school paradigm is glimpsed. For Bazarra and Casanova (2013), being able to characterise a smart school requires it to be identified with a way of using and transforming what happens on a daily basis, in order to create a smart future, rather than ceasing to propose new learning for new challenges. In this sense, Sánchez Povedano (2015) considered that in the formal educational sphere, this era has come to be characterised as the era of intelligence, where the school has to be aware of and prepared for such substantial changes. Factors such as globalisation, ICT involvement, the widening of learning spaces and lifelong learning, migratory movements in different countries, an extension of compulsory education, the heterogeneity of classrooms, and educational integration of diversity, as well as the loss of authority and teacher’s prestige, lead to a necessary rethinking of their competences by taking into account cognitive skills and attitudinal elements that shape them as professionals (Sánchez-Tarazaga 2016). Considering the European Commission report (2018), 8 competences exist (Figure 1). The different reports of international organisations (Eurydice 2002, 2013; OECD 2005, 2011, 2012; UNESCO-ILO 2000, 2009, 2012) establish a competency model for teachers that includes five essential dimensions of knowledge.

From these considerations, when contextualising the smart school paradigm, three fundamental interrelated aspects must be taken into account: The view of learners and their possibilities needs; the decision-making process of technical–pedagogical guidelines, introducing fundamental methodological changes; and organisational change (Sánchez Povedano 2015):

Within the demands that a smart school requires, Bazarra and Casanova (2013) provided an answer to the reason for this research article, with the following approach: Greater technical mastery in the professional sphere, as well as teacher training that enables a new professional profile in a continuous process of transformation. For Pogré (2012), it is crucial that teacher training rethinks the concept of teaching, moving away from the teaching–learning process as an accumulation of content and outlining the challenges facing teachers when it comes to proposing meaningful teaching processes that respond to the diversity of students in today’s classrooms. Gómez et al. (2022) agreed with this approach when considering the ongoing training of teachers as a basic strategy to respond to the challenges of contemporary schools through new methods, perspectives, and practices that reach the transformation of instructional processes, as well as a greater connection with the reality of students.

According to Pogré (2012), teacher training is a subject that has been approached with a scientific interest in recent decades, focusing on topics such as the need to redefine their practice, necessary changes in initial training, and the importance of professionalisation, but the author simultaneously argued that all of this work does not have an impact on training teachers whose task is to develop a practice in complex contexts. However, although there is still a long way to go, initiatives such as the “Future classroom Lab” project aim to create innovative spaces in which teachers can discover, explore, and investigate the essential components required for learning processes in this century (Gómez et al. 2022). It is clear that it is not a question of teacher training focused exclusively on professional competences, but rather of offering new tools that can encourage the entire educational community as a whole, giving way to new multidisciplinary teaching spaces. It would therefore lead to the discovery of other learning scenarios and modalities adapted to the change of mentality demanded by modern society.

Taking into account the need for changes in models, it is important to highlight the prevailing paradigm that characterises the so-called smart school. MacGilchrist et al. (2004) highlighted four characteristics or backbones within the aforementioned literature. The first of these is about accountability and student rights. The second focuses on leadership, management, and professional development. The third focuses on the teaching and learning process for students. Additionally, the fourth characteristic, which underpins the previous ones, is that of a learning organisation, understood as a school that is willing to learn and participate collaboratively in the development of its educational activity. In this respect, the main forms of continuous training chosen by teachers, according to the study by Nieto and Alfageme (2017), are courses (face-to-face or online), self-learning, and training in centres. However, it is also important to determine what content teachers tend to opt for in order to find out whether the training received responds to current needs. In short, it would also be useful to explore whether there are differences between the different generations of teachers when it comes to establishing and choosing their training actions and to check whether there is any impact on the assumption of this new paradigm of what teaching in schools should be.

Based on the above, there is a need to determine what kind of training experiences are being carried out by teachers in primary and secondary education and what the results of these experiences are for the benefit of their professional development as teachers under the paradigm of the smart school. To this end, the general objective of this study was to analyse the training activities carried out by primary and secondary school teachers over the last decade.

## 2. Materials and Methods

Taking into account the purpose of this research work, which sought to scientifically verify the evolution of teachers’ professional development towards the acquisition of the necessary competences under the smart school paradigm, it is considered that a systematic review methodology may constitute a relevant procedure for ascertaining the state of the art in this respect. A systematic review was conducted according to the recommendations of the PRISMA 2020 (Preferred Reporting Items for Systematic Review and Meta-Analysis) statement by Page et al. (2021).

To proceed methodologically, we started from the process established by Sánchez Meca and Botella (2010), in which the authors identified five stages for conducting systematic review and meta-analysis studies, which have been used successfully in the field of education by Monroy and Hernández Pina (2014) and Eiguren et al. (2022). The first of these corresponds to the statement of the research problem, as indicated in the previous section. Second, it was necessary to establish the selection criteria. In this case, we included studies with a qualitative, quantitative, or mixed design, written in Spanish, English, Catalan, or Portuguese, carried out from January 2012 to July 2022, and containing conclusive data, results, and measures related to training actions for teachers’ professional development, specifically in basic and secondary education. Analyses of studies developed in any global context were selected. Theoretical studies and narrative essays were excluded. Third, it was necessary to establish a measure of the quality of the selected analyses. This was achieved by following the guidelines offered by both the Caspe critical reading guide for qualitative studies (Cabello 2015) and the PEDro scale for quantitative studies (Sherrington et al. 2000), which have, again, been included in systematic review works in the field of education such as that of Martínez de Miguel et al. (2020), and which are scored on a minimum of 0 to a maximum of 10 points. Fourth, the search process was carried out by consulting different electronic databases. In the particular case described here, Google Scholar, ERIC, Scopus, Web of Science, and Dialnet were used. To reinforce the quality indicator of the selected studies, the ICDS value (a composite index of secondary diffusion) for the year 2021 of the journals provided by MIAR V.22 Live (Information Matrix for the Analysis of Journals) was taken into account. The values obtained were classified as having very high diffusion with values above 9.5, high diffusion with values ranging between 9.49 and 7.5, medium diffusion with values ranging between 7.49 and 6, and low diffusion with values below 6 points.

In contrast, and for their correct identification, the Boolean operators AND/AND and OR/OR were used, combining the following keywords in the title and abstract of the articles, in both English and Spanish: “educational personnel training or teachers training or teacher education and beginner teachers,” “educational personnel training or teachers training or teacher education and senior teachers,” and “educational personnel training or teachers training or teacher education and retired teachers.”

**Figure 2:**
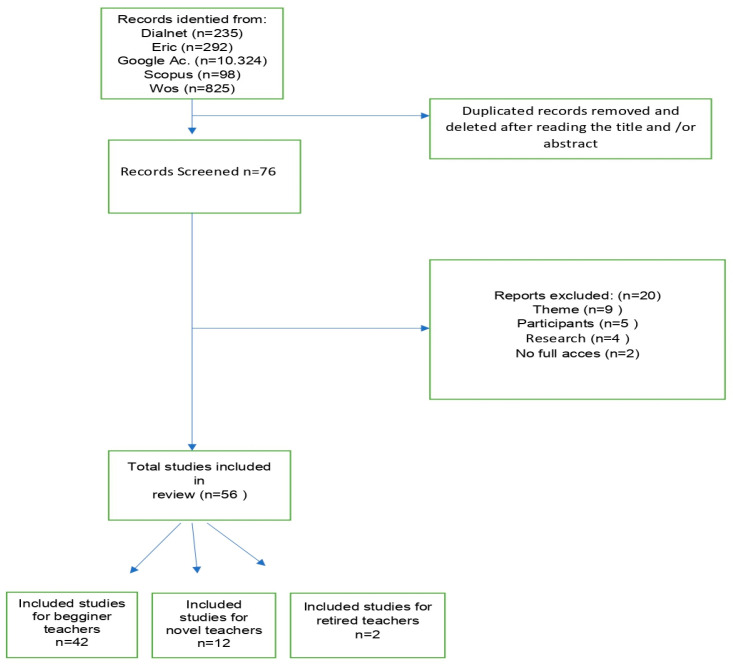
Selection flow of the articles included in the systematic review.

In order to better understand the process, a flow chart is provided in Figure 2.

The fourth stage was the coding of the research and the extraction of the information, once the abstract, title, and keywords of all of the articles had been read. Once the reading was completed, those articles that met the defined criteria were selected, and for this purpose, the PICOS strategy was taken into consideration, as recommended for the reporting of systematic reviews in some works such as that of Rubio et al. (2018). Based on this, we proceeded to develop a manual of characteristics and a registration protocol for the extraction of data from the articles. For this purpose, a database was created with the variables collected. The variables extracted from each of the studies were authors, title, journal, year, keywords, country, objective, subject, methodology, sample size, teacher generational typology, type of training, and stage of training. The analytical coding process was carried out by three researchers, who independently entered the data into the data coding database. For this process, the indicators used for the selected variables were taken into account. The information was studied following a qualitative analysis of the content of the studies, supported by the calculation of frequencies and averages.

Finally, the results of the selected articles were grouped according to the generation variable. In this sense, three groups were identified: Novel, veteran, and retired. The reason for differentiating by generations corresponds with trying to identify whether there are differences in terms of the type of training content, according to the professional development of young, veteran, and retired teachers.

## 3. Results and Discussion

The results were written following the proposal of Menéndez et al. (2022). Before diving into the obtained data, it is necessary to reflect on the characteristics of the collected articles and the main selected variables. This can provide valuable insights into the distribution and reach of the selected publications, as well as the specific audiences they target. By analysing this information, researchers can gain a better understanding of the overall landscape of the publications in question and their potential impact on their fields of study. This aspect is an essential element in a systematic review, but it is also very necessary in order to find out whether the training actions developed for teachers are being carried out with the scientific rigour that would be required to analyse the state of the question. It has been noted in the search process that there are numerous training experiences in this respect, but fewer are organised around a research study. Specifically, in the analysis carried out in this case, it is clearly identifiable that the type of studies chosen in this research topic is the qualitative methodological approach, which is very marked in an ethnographic perspective and centred on individual testimonies. This is discordant with the study by Bautista and Ortega-Ruíz (2015), which indicated that the predominance of studies on teachers’ professional development corresponds to the evaluation of programmes. In the specific case of the research presented herein, it should be added that the focus was more on identifying factors and detecting needs. Furthermore, it was observed that in this topic of analysis, there were no differences between the generational group with which the study had been carried out, although it can be seen that the greatest number of studies were carried out by the youngest generational group.

**Figure 3:**
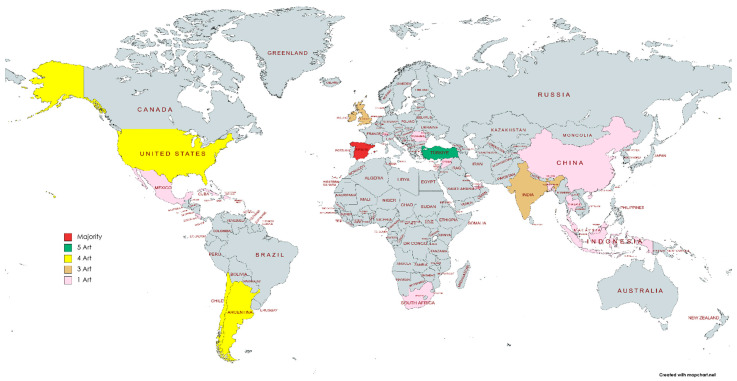
Distribution map of the selected articles.

From the perspective of the geographical distribution of the selected publications, Ibero-American countries had the highest production of articles relating to teacher training studies or teacher training in the school teacher training sphere. Spain was the country with the highest number of references written on the subject (17 articles). In terms of the number of articles selected by country, Spain was followed by Turkey (five articles) and the United States (four articles). All continents were represented, with the exception of Australia. In Europe, the United Kingdom stood out (three articles), followed by Switzerland (one article) and Romania (one article). Among the Latin American countries, Argentina and Chile stood out (four articles), followed by Colombia, Cuba, Mexico, the Dominican Republic, and Costa Rica (one article each). On the African continent was South Africa (one article). In Asia, articles were published by India (three articles), Bangladesh (one article), and China (one article). Middle Eastern countries included Lebanon (one article), while in Southeast Asia, they included Thailand, Indonesia, Malaysia, and Thailand (one for each country). Meanwhile, in the Arabian Peninsula was the United Arab Emirates (one article). No items were found from Russian or Korean sources (Figure 3).

In relation to the type of journals where the articles were published, the articles selected for the group of new teachers totalled 42 articles and were published in a wide variety of journals and topics. Most notably, 84% (*n* = 35) were published in specialised educational studies journals and 16% (*n* = 7) in non-specialised journals. Among them, their areas of interest were varied, with technologies, physical education, and science standing out in particular. Among those specific to the area of education, 31% (*n* = 12) belonged to high-impact publications. Two studies from national education centres in the USA were also selected.

Concerning the group of senior teachers, a total of 12 articles were selected, corresponding mostly to specialised education journals (83.3%; *n* = 10) and only 16.6% (*n* = 2) belonged to non-specific journals. In the group of specialised journals, 50% (*n* = 5) belonged to high-impact journals.

As far as the group of retired teachers is concerned, two articles were selected, both (100%) belonging to specialised and high-impact journals.

Regarding the quality of the selected articles, all of the quantitative studies were observational studies, with no experimental studies having been carried out. Among the studies with large samples, with more than one thousand participants, the study by Manso and Garrido (2021) with *N* = 1148 teachers stood out, but among all of the related studies, the study by Álvarez (2016) with *N* = 2656 stood out. With regard to the results obtained using the PEDro Guide for quantitative studies, the value of the mode was 4/10. Meanwhile, regarding the results obtained after checking the qualitative articles using the CASPe guide, the results of the value of the mode were 8/10.

Among the publications with the highest impact indicators, internationally published articles were found in: “Revista de Educación” (Fecyt, quartile 1), “Education Science” (Cite Score 2.9), and “Revista Complutense de Educación” (Cite Score 2.6). It should also be noted that more than 50% of the articles selected for the review had very high diffusion in the journals in which they were published (ICDS > 9.5).

On the subject of the predominant type of methodology found in the articles referring to new teachers, qualitative methodology was predominant in 21 articles (48.8%), with case studies and content analysis through semi-structured interviews, accompanied by autobiographies and personal biographies. This was followed by quantitative methodology in 17 articles (41.8%) and exploratory descriptive studies where survey methodology through questionnaires predominated. Finally, a mixed methodology was found in three publications (9.3%), which were the studies of Roig and Urrea (2020), Alghamdi (2021), and Aliyyah et al. (2020). Regarding the methodology used in the studies with veteran teachers, seven studies (61.1%) were developed using qualitative methodology, four with quantitative methodology (33.3%), and one mixed methodology study (5.5%). With regard to the retired population, two studies were carried out using a qualitative methodology (100%), approached through the teaching life testimonies of 325 teachers (Parra et al. 2020), and another was carried out using the interview methodology.

Differences were found with respect to the total in the thematic indexes between the theoretical studies developed for the identification of factors (*n* = 25; 43.3%), those that need a diagnosis (*n* = 18; 33.3%), and those more practical studies that propose actions (*n* = 13; 23.3%). It is in the latter that mentoring processes stood out as the most frequently mentioned activity (Khanam et al. 2022).

**Figure 4:**
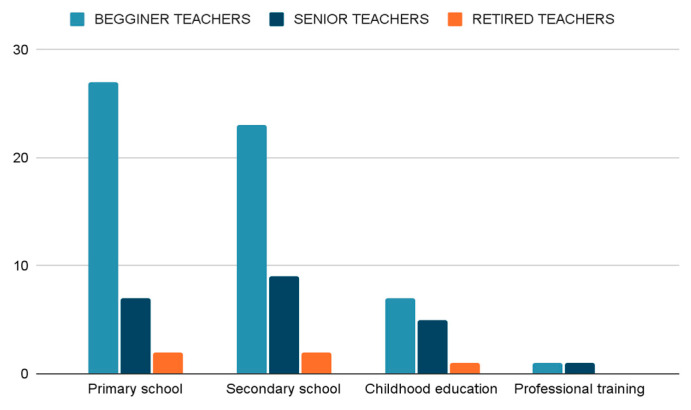
Selected studies according to educational context and teachers.

With reference to levels of education, the studies were not exclusively limited to one level of education. However, there was a clear pattern. In this respect, it is worth noting that the highest number of studies was in primary education with new teachers and the lowest was among retired vocational teachers, as shown in Figure 4.

**Figure 5:**
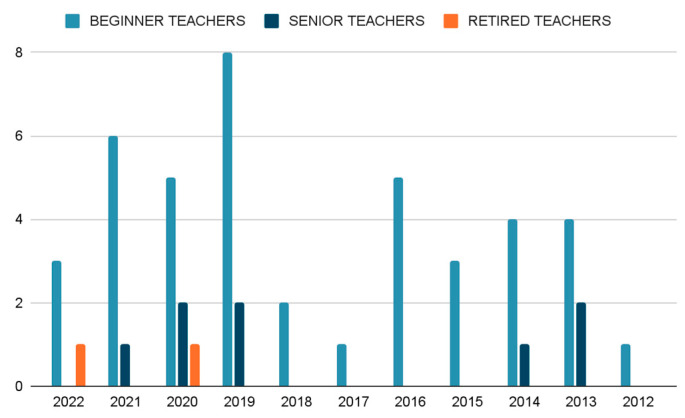
Year of publication of the selected articles.

In terms of the year of publication (Figure 5), no standardised pattern could be observed, although it is true that an increasing number of publications on retired teachers have been published in recent years.

**Figure 6:**
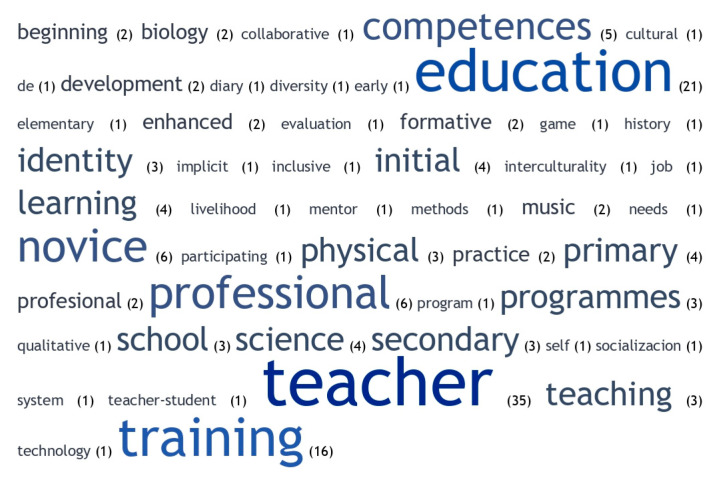
Word cloud for studies of new teachers.

In the articles analysed referring to new teachers, the concepts most commonly used as keywords were training, which, together with competences (Suciu and Mâţă 2014), identity, and professional (Cortés et al. 2014), would be the most appropriate in this context associated with professional development, highlighting the area of primary education (Figure 6). In contrast, words such as education, learning, teacher, programmes, and school appeared, indicating direct links with education. These were also words that refer to educational elements or disciplines such as physics, biology, and physical education. Other concepts of interest that stood out were research and professional life.

**Figure 7:**
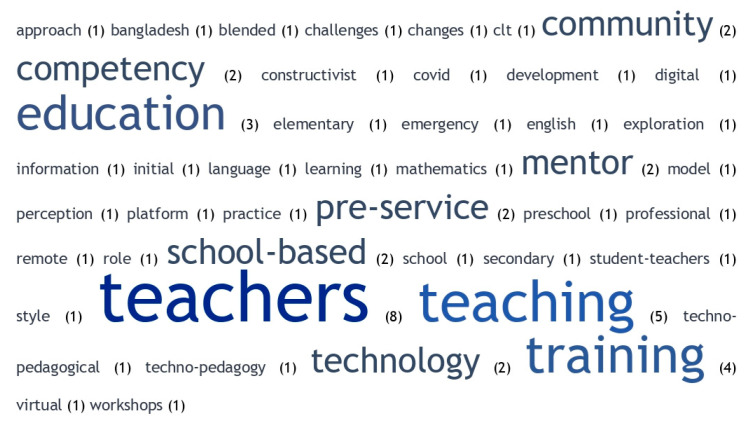
Word cloud among senior teachers.

As far as veteran teachers are concerned, there were certain coincidences, such as the words training or competences as the most repeated words, but there were also other different words, such as technology (Trust et al. 2016) (Figure 7).

**Figure 8:**
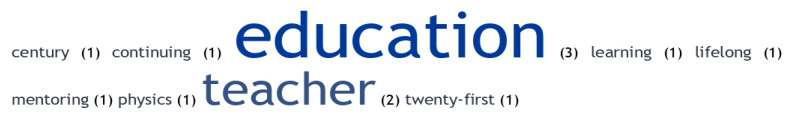
Word cloud on retired teachers.

As for the retired group, although training continued to be the main word, terms referring to the concept of lifelong learning or lifelong learning appeared (Domínguez et al. 2015), as well as mentoring (Figure 8).

With the intention of interpreting the results obtained, resorting to seminal research on the subject of study of the review carried out as a point of reference for the discussion, it is worth noting that there is strong evidence that a culture of learning among teachers is an important factor in the quality of teaching in a smart school (Preston et al. 2017). A culture of learning involves teachers being committed to their professional development and being open to continuous learning and improvement (Kardos and Johnson 2007). This can lead to more effective teaching and a more positive learning environment for students (Azorín et al. 2022). Additionally, when teachers, support each other and share their knowledge and skills, they can improve the quality of teaching (Vallejo Ruiz et al. 2021). In short, a culture of learning among teachers is an important factor in the quality of teaching in an educational centre (Negrín-Medina et al. 2022; Portela and Nieto 2021). According to the data obtained regarding the teaching competencies that are being developed in the teacher training of school teachers, and the level at which they are located, currently, it is worth noting that different competencies are being developed, such as the ability to design and plan teaching sequences, the ability to use different teaching methods and resources, communicative competence, and the ability to work in teams. There is also a greater emphasis on training in digital competencies and the incorporation of new technologies in the classroom.

**Figure 9:**
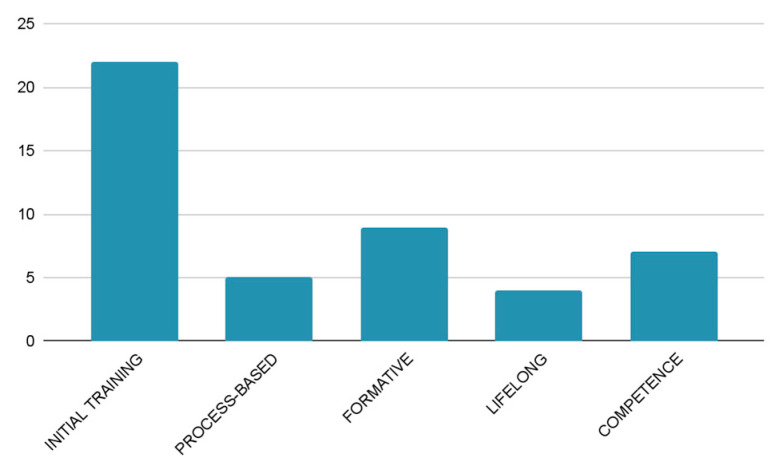
Training of new teachers.

In order to be able to synthesise the typology and characterisation (Figure 9) of the studies on teacher training and the competences that are implemented, it should be noted that from the group of new teachers (Nomlomo and Sosibo 2016), adjustments were observed in their training courses to the new needs arising from the globalised society (Rosso and Olivieri 2019) in terms of foreign language and information society competences with the use of ICT (Rahman and Ahmed 2019), changes in evaluation processes, including teacher satisfaction and its transfer to the classroom, the personalisation of training pathways based on teachers’ professional competences (Seker and Deniz 2016), and modifications in the management of training courses to adapt to professional needs and diversity (Vidiella and Larrain 2015). The studies focused on ICT, professional identity (Aristizabal 2019; Souto et al. 2020), learning experiences, stress management, teacher support and motivation, evidence-based practices, challenges, and technical teaching.

The majority of senior teachers underwent in-service training focusing on mentoring, pedagogical guidance, technology integration, professional identity, and technical skills (Sainz and Ozaeta 2013). This work showed that this indicator can be useful as a marker of professional development within the paradigm known as “smart schools,” related to the studies of Rodríguez Hontanilla (2015), Arrieche (2018), and Mota (2020).

Finally, retired teachers were trained within the paradigm of lifelong learning on the psychological and pedagogical aspects of mentoring and tutoring young teachers.

If we look at the contributions of teacher training studies to the field of smart schools, one of the initial aspects to highlight is that the content of the training actions in which the research carried out corresponds to the competences inherent to the requirements of a smart school. Although it is true that ICT occupies an important percentage as a necessary competence (Prieto et al. 2021), the main purpose of the smart school paradigm is to educate citizens who are characterised by active, self-directed learning, provide them with security, and require them to be socially committed and competent in the different cognitive, emotional, social, and technological dimensions (Bautista and Ortega-Ruíz 2015; Elli and Ricafort 2020). These types of changes necessarily influence teachers’ professional development and were not globally reflected in the analysis carried out.

In this context, it is important to promote the use of training and the use of tacit intergenerational collaborative knowledge, which favours the emergence of “other learning” generated as a result of accumulated professional experience (Ergunay and Adiguzel 2019), and its combination with more innovative learning, techniques, and methods, where information and knowledge flow from the younger generations to the older ones, and vice versa. Regarding the younger teachers, competencies are identified in the training carried out tending towards the management of coexistence and social-relational competencies (knowing how to be), as well as didactic competence (knowing how to do). Among veteran teachers, the competences related to work teams stand out (competence of knowing and knowing how to do what). In retired teachers, intra- and interpersonal skills (knowing) were identified.

In this way, not only will teaching quality and educational excellence increase but it will also allow for a more contemporary conception and perspective of professional development that is adjusted to the needs, requirements, and problems faced by the 21st-century educator (Lombardi and Abrile 2009; Bayar 2014; Butt and Farooq 2019).

A large part of the data collected in this study came from research carried out with *novice teachers.* Most selected articles regarding the youngest teachers identify skills as part of the developed training, which aims at managing coexistence and social-relational abilities (knowing how to be), as well as didactic ones (knowing how to do). In this context, we found aspects that refer to psychological aspects (Herrera et al. 2019), such as work on the expectations of graduate teachers (Rosso and Alcalá 2016), the reinforcement of creativity in initial training (Arriaga et al. 2021), or the need pointed out in the study by Vicente and Gabari (2019) emphasising the importance of preventing emotional exhaustion syndrome secondary to socio-affective and psycho-emotional components derived from the profession of dedication and service to students. It is necessary to take into account the teacher’s identity (Errobidart 2015) and the threats posed by the profession (loneliness, de-qualification, identity crisis, etc.), which González Calvo (2013) mentioned.

In contrast, transferable strategies and methodologies were observed, where Hinojosa et al. (2020) emphasised the need to reinforce skills to solve emerging problems, and the scarce management of administrative and technical aspects. Other authors expressed specific training needs, such as educational inclusion (Fernández et al. 2021). Additionally, other training needs were identified, including training in aspects of legal responsibility and ethical behaviour, as well as school coexistence, individual attention to students with behavioural problems and aggression (disruptive behaviour), the resolution of coexistence problems in the classroom, conflict resolution with the support of parents, and conflict resolution in the school context (Cordero et al. 2021). In this sense, the study by Jara (2020), in the context of didactics in the area of science (chemistry, physics, and biology), pointed to the need to improve the presence of reference competences in their training. Taking this into account, there seems to be poor pedagogical training (dissociation theory/practice, student characteristics and their attitude to the subjects they teach, and difficulties in finding classroom strategies that motivate students or the demands of the institutional context), as pointed out by Rosso and Alcalá (2016).

The results showed that the fundamental core was ICT in the first place, as indicated. In this sense, the study carried out by Sangrá et al. (2019) coincided with the systematic review carried out with regard to the professional development of primary school teachers in highlighting technologies as one of the crucial aspects to be taken into account in training actions (Kamışlı 2019). In this core area, important generational differences were noted, because there were notable generational differences in training at the outset so ICT is more in demand among the older generations. Penalva et al. (2013), for their part, pointed out that at a methodological level, novice teachers encourage teamwork, a practice that is not used by experts. Álvarez (2016), in contrast, argued that secondary school teachers in Spain do consider ICT to be very important as a necessary part of initial and continuing teacher training, but it is precisely the youngest and also the most inexperienced teachers who value them to a lesser extent. This is also corroborated by international research (Al-Derbashi and Abed 2017). The study by Roig and Urrea (2020) pointed out in its conclusions the lack of formal opportunities for intercultural collaborative training.

The studies on the subject of support for new teachers are noteworthy. Corrales et al. (2022) stated that it is essential to design a plan in view of the learning outcomes for professional initiation. Different technical documents at the international level pointed to the experiences of resident teachers in the American context (Silva et al. 2014; Zweig et al. 2015). At the national level, this need to implement and improve the accompaniment of novice teachers in Spain was evident in the study by Manso and Garrido (2021).

From a more epistemological perspective, Roig and Urrea (2020) highlighted that the principle of collaboration among the teaching staff itself is not fully valued as a strategy for learning, but rather as a resource for institutional functioning, which is why it is considered to be of interest as a possibility for improvement. However, in the study developed by Azcárate and Cuesta (2012), factors that facilitated the change in the practices, ideas, and attitudes of teachers are noted: The recognition of their own practical problems, the positive attitude to address them, and the dynamics of collaboration. In the same way, Ravanal (2019) argued that teaching must be installed in paradigms that are more autonomous for the student and that promote higher-order learning. It is therefore imperative to distance ourselves from teaching models focused on memorisation and the reproduction of knowledge.

With regard to higher education institutions, Zapatero et al. (2018) pointed out the possibility of strengthening initial or competency-based teacher training (Noom 2013), as well as placing greater emphasis on continuous training (Shaaban and Imane 2018) and moving away from the traditional way of assessment in the classroom (Cañadas and Santos-Pastor 2021), which is more memoristic and finalistic.

In relation to the research carried out by veteran teachers, the competences related to work teams stand out (competence of knowing and knowing how to do what), and it is interesting to highlight the idea of Carrió et al. (2013) for the creation of a community of blended learning teachers with different profiles, although the results of their study showed that they are more committed to face-to-face teaching with a lower degree of involvement on the part of veteran teachers compared to novice teachers. It is not surprising, therefore, when it is stated that for adequate professional development, mentor teachers are important in the school and have a great influence on teaching practice (Baartman 2020). In pursuit of the same objective, the influence of educational technology has been studied (Özdemir 2016). Some studies claimed, as in the case of Lau and Yuen (2013), for mathematics teachers, that training in educational technology increases the effectiveness of senior mathematics teachers but does not change their beliefs about the educational benefits of using technology after the training. This was also supported by the study of Umar and Yusoff (2014), who stated that senior teachers use ICT less than younger teachers. Additionally, from a broad perspective of analysis, it is considered interesting to pay attention to institutional support at the level of different education laws and the perception that contemporary legislation has regressed in terms of support for teachers, as reflected in the study by Webber and Hardwell (2019). This is in addition to encouraging school management to increase staff training and to develop their competencies, based on studies such as that developed by Abdul (2015). Finally, a last aspect of interest pointed out by senior teachers is the differences that can be found in schools, depending on their nature and socioeconomic context, whereby it can be argued that well-designed induction and mentoring programmes in rural or under-resourced areas could be another strategy to increase teacher retention, based on the data obtained in the study by Ozoglu (2015).

Finally, in the studies on retired teachers, intra and interpersonal competences (know) were identified, and it is worth highlighting Parra et al. (2020), where they recommended the participation of younger teachers by generating intergenerational contexts in which they can deliberate freely and giving them an essential starting point for reflection in the early stages of their professional career. Similarly, perceptions of the training requirements not only include being a good teacher but also specify, as per the study of Cottle (2022), remaining in the profession. However, perhaps the most substantial aspect of this perspective is that according to the data collected from the studies, there are significant differences according to generation and year of birth and a need for training in ICT, training in practical methodologies, cultural training, and training in educational psychology.

## 4. Conclusions

The starting point of this study was the need to better understand the training carried out by primary and secondary school teachers, and whether it contributes to the professional development of teachers from the perspective of the paradigm of intelligent schools. In the training of school teachers, different pedagogical strategies are being developed that can be transferred to the configuration of smart schools. For example, active and participatory methodologies such as cooperative learning or project-based work are being promoted, which foster autonomous learning and collaboration among students. The incorporation of educational technologies and training in digital competencies is also being worked on, which contributes to the creation of more flexible and adaptive educational environments.

Teacher training is a key aspect in the professional development of teachers within the paradigm of intelligent schools and involves a planned and systematic approach to continuously improve teachers’ skills and knowledge in order to provide quality education to their students. This is because intelligent schools focus on providing students with a high-quality education that allows them to develop valuable skills and knowledge in a digital and changing environment. Teachers must be trained in the use of technology and innovative teaching methods in order to implement them in the classroom and promote meaningful and tailored learning. Additionally, teacher training also plays a key role in creating a learning and collaborative culture in the school, which is essential in the development of an intelligent school. In addition to training in the use of educational technology and innovative teaching methods, teacher training in the context of intelligent schools must also address other important aspects, such as attention to diversity and the inclusion of students, collaboration and teamwork, and management of change and innovation in the classroom. This is essential to ensure that teachers are prepared to face the challenges of education in the 21st century and can contribute to the success of students in an increasingly complex and changing environment. The systematic review carried out herein brought together the most substantial international studies carried out on school teacher training.

In relation to the contents of training experiences according to educational levels and generational groups, it was found that training activities are mainly carried out in primary education and by younger teachers, which could lead to future research to analyse why, as the years go by, the number of training activities to be carried out by teachers decreases. However, it is the concepts of mentoring and lifelong learning that are most closely associated with the concept of professional development. To summarise, it seems logical that due to the dynamics of the professional career itself, it is the younger teachers who should be the focus of research to encourage this process, and the older generations of teachers, both retired and veteran, who should carry out the mentoring, induction, and tutoring processes for new teachers.

The question must be asked: Are schools ready for a paradigm shift towards an intelligent culture? There may be advantages and disadvantages to this attitude. On the positive side, for example, it demonstrates that this type of school is flexible, receptive to change, and willing to improve—the most valuable precondition that will determine its progress. On the negative side, however, this can foster a culture of dependency in which schools look for models or formulas that they can apply in a mechanistic way, regardless of their own particular context and culture. This, in turn, encourages consultants and external agencies to offer simple solutions to what are often very complex problems.

Furthermore, the use of this knowledge will help future teachers and educational administrators to plan and establish new possibilities for the enrichment of the teaching profession and can be focused on a school of the future, being more democratic and collaborative, and with an intergenerational perspective, taking advantage of the potential and possibilities for knowledge exchange in the educational centres themselves.

## Data Availability

Not applicable.

## References

[B1-jintelligence-11-00001] Abdul Nina Lufti (2015). The lived-through experience of the senior teacher: A closer look at a middle management and leadership position in Bahraini public schools. Cogent Education.

[B2-jintelligence-11-00001] Al-Derbashi Khaled Younis, Abed Osama H. (2017). The Level of Utilizing Blended Learning in Teaching Science from the Point of View of Science Teachers in Private Schools of Ajman Educational Zone. Journal of Education and Practice.

[B3-jintelligence-11-00001] Alghamdi Ahmad Saad (2021). Training Teachers to Implement Evidence-Based Practices Specifically Designed for Students with Autism Spectrum Disorder. Training.

[B4-jintelligence-11-00001] Aliyyah Rusi Rusmiati, Rachmadtullah Reza, Samsudin Achaman, Syaodih Ernawulan, Nurtanto Muhammad, Tambunan Anna (2020). The perceptions of primary school teachers of online learning during the COVID-19 pandemic period. A case study in Indonesia. Online Submission.

[B5-jintelligence-11-00001] Álvarez Juan Francisco (2016). ICT training for secondary school teachers in Spain. An analysis from the teachers’ perception. Universitas Tarraconensis. Revista de Ciències de l’Educació.

[B6-jintelligence-11-00001] Aristizabal Andrea (2019). Strengthening the teacher’s professional identity in the personal sphere of the teacher. Tecné, Episteme y Didaxis.

[B7-jintelligence-11-00001] Arriaga Crisitna, Eguiluz Baikune de Alba, Ibarmia Gorka (2021). Creativity in the teacher training process. An approach to creative experiences in new music teachers. Arsseduca.

[B8-jintelligence-11-00001] Arrieche Marilin Pastora (2018). Teaching Management in the Context of Venezuelan Primary Education. Revista Scientific.

[B9-jintelligence-11-00001] Azcárate María Pilar, Cuesta Josefa (2012). Factors that facilitate change in new secondary school teachers. Revista de Educación.

[B10-jintelligence-11-00001] Azorín Cecilia, Antonio Portela, Miguel Nieto Jose, Begoña Alfageme María (2022). Professional relationships both within and outside the school: Barriers and opportunities from an intergenerational perspective. Journal of Professional Capital and Community.

[B11-jintelligence-11-00001] Baartman Nomakhaya (2020). Challenges experienced by school-based mentor teachers during initial teacher training in five selected schools in Amathole East District. e-BANGI.

[B12-jintelligence-11-00001] Bautista Alfredo, Ortega-Ruíz Rosario (2015). Teacher professional development: International perspectives and approaches. Psychology, Society and Education.

[B13-jintelligence-11-00001] Bayar Adem (2014). The Components of Effective Professional Development Activities in Terms of Teachers’ Perspective. Online Submission.

[B14-jintelligence-11-00001] Bazarra Lourdes, Casanova Olga (2013). Directivos de Escuelas Inteligentes: What Profile and Skills Does the Future Demand?.

[B15-jintelligence-11-00001] Butt Khadija, Farooq Muhammad Shahid (2019). Effect of induction training program on teachers’ effectiveness at elementary school level in Punjab. Pakistan Journal of Education.

[B16-jintelligence-11-00001] Cabello Juan Bautista (2015). Critical Reading of Clinical Evidence.

[B17-jintelligence-11-00001] Cañadas Laura, Santos-Pastor María Luisa (2021). Formative assessment from the perspective of novice teachers in primary and secondary physical education classes. Educare Journal.

[B18-jintelligence-11-00001] Carrió Mar, Soria Vanessa, Costa Marcel, López Silvia (2013). Innovaula. Creating a community to innovate in science classrooms. Enseñanza de las Ciencias: Revista de Investigación y Experiencias Didácticas. Nº Extra.

[B19-jintelligence-11-00001] Cordero Graciela, García Jihan, Rivera Karen Patricia, Figueroa Karla, Gastelum Guadalupe, Almaraz Silvia (2021). Diagnosis of training needs of novice elementary school teachers in Baja California. Revista CPU-e.

[B20-jintelligence-11-00001] Corrales Orlando, Valdés Mercedes, Monteagudo Jose Francisco (2022). Actions for the strengthening of in-service training in novice Physical Education teachers. Podium. Journal of Science and Technology in Physical Culture.

[B21-jintelligence-11-00001] Cortés Pablo, Leite Analía, Rivas José Ignacio (2014). A narrative approach to professional identity in novice teachers. Tendencias Pedagógicas.

[B22-jintelligence-11-00001] Cottle Daniel (2022). Harnessing the potential of recently retired physics teachers to mentor new physics teachers. Physics Education.

[B23-jintelligence-11-00001] Domínguez Jose, Calvo Jesús, Vazquez Elia (2015). Evaluation of permanent teacher training: Approach of results. Evaluation of permanent teacher training: Approach of results. Journal of Studies and Research in Psychology and Education.

[B24-jintelligence-11-00001] Eiguren Amaia, Berastegi Naiara, Correa Jose Miguel (2022). Combating the generation gap: Systematic review of intergenerational experiences carried out in the school environment. Revista de Investigación Educativa.

[B25-jintelligence-11-00001] Elli María Cristina, Ricafort Jhonner (2020). Competencies of Grade VI Teachers in Technology and Livelihood Education (TLE). Online Submission.

[B26-jintelligence-11-00001] Ergunay Onur, Adiguzel Oktay Cem (2019). The first year in teaching. Changes in beginning teachers’ visions and their challenges. Qualitative Research in Education.

[B27-jintelligence-11-00001] Errobidart Analía Elisabet (2015). The construction of identities of novice teachers in their labour insertions. A contextualised study in present-day Argentina. Reire.

[B28-jintelligence-11-00001] European Commission (2018). Council Recommendation on Key Competences for Lifelong Learning.

[B29-jintelligence-11-00001] European Commission/EACEA/Eurydice (2018). The Teaching Profession in Europe: Access, Progression and Support. Eurydice Report.

[B30-jintelligence-11-00001] Eurydice (2002). Key Issues of Education in Europe. The Teaching Profession in Europe: Profile, Trends and Problems.

[B31-jintelligence-11-00001] Eurydice (2013). Key Data on Teachers and School Leaders in Europe.

[B32-jintelligence-11-00001] Fernández María Eugenia, Rey Carmen, D’Ottavio María Eugenia (2021). Training primary school teachers in inclusive education in Rosario (Argentina). RAES.

[B33-jintelligence-11-00001] Gómez Melchor, Alameda Alberto, Poyatos Cesar, Ortega Pablo Javier (2022). The classroom of the future. A project for the pedagogical redefinition of educational centres. Revista Interuniversitaria de Formación del Profesorado.

[B34-jintelligence-11-00001] González Calvo Gustavo (2013). The struggle of a novice Physical Education teacher for the recognition of his profession narrated from an autobiographical perspective. Agora for Physical Education and Sport.

[B35-jintelligence-11-00001] González Fernando y, Escudero Jacinto, Jímenez En A. (2017). Las competencias sociales y cívicas. Las competencias educativas e innovación.

[B36-jintelligence-11-00001] Herrera Lucía, Perandones Teresa María, Sánchez Laura del Carmen (2019). Personal strengths and teaching effectiveness. International Journal of Developmental and Educational Psychology.

[B37-jintelligence-11-00001] Hinojosa Claudio, Hurtado Macarena, Magnere Paula (2020). Novice physical education teachers. Perceptions of their teacher training based on performance in the school system. Retos.

[B38-jintelligence-11-00001] Jara Roxana (2020). The performance of novice science teachers. The professional competencies they develop during the first years of professional practice. Pensamiento educativo. Revista de Investigación Educacional Latinoamericana.

[B39-jintelligence-11-00001] Jerrim John, Sims Sam (2019). The Teaching and Learning International Survey (TALIS). https://assets.publishing.service.gov.uk/government/uploads/system/uploads/attachment_data/file/919064/TALIS_2018_research.pdf.

[B40-jintelligence-11-00001] Kamışlı Halil (2019). On primary school teachers’ training needs in relation to game-based learning. International Journal of Curriculum and Instruction.

[B41-jintelligence-11-00001] Kardos Susan M., Johnson Susan Moore (2007). On their own and presumed expert: New teachers’ experiences with their colleagues. Teachers College Record.

[B42-jintelligence-11-00001] Khanam Adila, Butt Intazar Hussain, Batool Hifsa (2022). Measuring the Effectiveness of Induction Training for Elementary School Teachers in Punjab. Journal of Elementary Education.

[B43-jintelligence-11-00001] Krischesky Gabriela, Murillo Francisco Javier (2018). Teacher collaboration as a learning factor and promoter of improvement. A case study. Educación XXI.

[B44-jintelligence-11-00001] Lau Wilfred Wing Fat, Yuen Allan Hoi Kau (2013). Educational technology training workshops for mathematics teachers: An exploration of perception changes. Australasian Journal of Educational Technology.

[B45-jintelligence-11-00001] Lombardi Graciela, Abrile María Inés, Velaz Consuelo, Vaillant (Coord) Denise (2009). Teacher education as a system. From initial training to professional development. Aprendizaje y Desarrollo Profesional Docente.

[B46-jintelligence-11-00001] MacGilchrist Barbara, Reed Jane, Myers Kate (2004). The Intelligent School.

[B47-jintelligence-11-00001] Manso Jesús, Garrido Rocío (2021). Those who are just starting out. The pending challenge of accompanying new teachers. Revista del profesorado.

[B48-jintelligence-11-00001] Martínez de Miguel Silvia, Salmerón Juan Antono, Abellán Pedro. Moreno (2020). Educational innovation in the degree of Social Education in Spanish universities. A systematic review. Educar.

[B49-jintelligence-11-00001] Menéndez David, Urbina Santos, Forteza Dolores, Rodríguez Alejandro (2022). Contributions of futures studies to education: A systematic review. Comunicar.

[B50-jintelligence-11-00001] Monroy Fuensanta, Hernández Pina Fuensanta (2014). Factors influencing university learning approaches. A systematic review. Educación XX1.

[B51-jintelligence-11-00001] Mota Jhoel (2020). Effective Decision Making from the Context of Smart Organizations in Primary Schools. Scientific Journal.

[B52-jintelligence-11-00001] Negrín-Medina Miguel Ángel, Bernárdez-Gómez Abraham, Portela-Pruaño Antonio, Marrero-Galván Juan José (2022). Teachers’ perceptions of changes in their professional development as a result of ICT. Journal of Intelligence.

[B53-jintelligence-11-00001] Nieto Jose Miguel, Alfageme Begoña (2017). Teacher training approaches, methodologies and activities. Revista de Currículum y Formación del Profesorado.

[B54-jintelligence-11-00001] Nomlomo Vuyokazi, Sosibo Lungi (2016). From theory to practice: Beginner teachers’ experiences of the rigour of the Postgraduate Certificate in Education programme. Perspectives in Education.

[B55-jintelligence-11-00001] Noom Sripathum (2013). English-Teaching Problems in Thailand and Thai Teachers’ Professional Development Needs. English Language Teaching.

[B56-jintelligence-11-00001] OECD (2005). The Definition and Selection of Key Competences: Executive Summary.

[B57-jintelligence-11-00001] OECD (2011). Building a High-Quality Teaching Profession: Lessons from around the World.

[B58-jintelligence-11-00001] OECD (2012). Preparing Teachers and Developing School Leaders for the 21st Century: Lessons from around the World.

[B59-jintelligence-11-00001] Özdemir Muhammet (2016). An Examination of the Techno-Pedagogical Education Competencies (TPACK) of Pre-Service Elementary School and Preschool Teachers. Journal of Education and Training Studies.

[B60-jintelligence-11-00001] Ozoglu Murat (2015). Teacher allocation policies and the unbalanced distribution of novice and senior teachers across regions in Turkey. Australian Journal of Teacher Education.

[B61-jintelligence-11-00001] Page Matthew, McKenzie Joanne, Bossuyt Patrick, Boutron Isabelle, Hoffmann Tammy, Mulrow Cynthia, Shamseer Larissa, Tetzlaff Jennifer, Akl Elie, Brennan Sue (2021). The PRISMA 2020 statement: An updated guideline for reporting systematic reviews. Systematic Reviews.

[B62-jintelligence-11-00001] Pamies Marcial, Cascales Antonia, Gomariz Mari Ángeles (2022). Association between conditioning factors of the transfer of lifelong learning and the application of learning in the training programmes of Pre-school and Primary Education. Revista Electrónica Interuniversitaria de Formación del Profesorado.

[B63-jintelligence-11-00001] Parra Gabriel, Navarro Ana Belén, Rubio Francisco Javier, Martín Bienvenido (2020). Teaching-life Histories: An Analysis of Initial and Continuing Training for Twenty-first century Teachers. ESE. Education Studies.

[B64-jintelligence-11-00001] Penalva Antonia, Hernández Mari Ángeles, Guerrero Catalina (2013). Effective teacher management in the classroom. A case study. Revista Electrónica Interuniversitaria de Formación del Profesorado.

[B65-jintelligence-11-00001] Pogré Paula (2012). Teacher education today: What do prospective teachers need to understand?. Perspectiva Educacional, Formación de Profesores.

[B66-jintelligence-11-00001] Portela Antonio, Torres Ana, Salmerón Juan Antonio, De Miguel Silvia Martínez, Dorczak Roman, Portela Antonio (2020). Generational diversity. Relevance for professional collaboration in organizational and educational contexts. Generational Diversity and Intergenerational Collaboration among Teachers. Perspectives and Experience.

[B67-jintelligence-11-00001] Portela Antonio, Nieto Jose Miguel (2021). Retired and veteran teachers also matter. El diario de la Educación.

[B68-jintelligence-11-00001] Preston Courtney, Ellen Goldring, Edward Guthrie J., Russell Ramsey, Jason Huff (2017). Conceptualizing Essential Components of Effective High Schools. Leadership and Policy in Schools.

[B69-jintelligence-11-00001] Prieto Jorge Manuel, Revuelta Francisco Ignacio, Pedrera María Inmaculada (2021). Secondary school teachers self-perception of digital teaching competence in spain following COVID-19 confinement. Education Sciences.

[B70-jintelligence-11-00001] Rahman Mehadi, Ahmed Rokiba (2019). The Effect of Teachers’ Training in Secondary English Teachers’ Practice of Communicative Language Teaching (CLT) in Bangladesh. Online Submission.

[B71-jintelligence-11-00001] Ravanal Eduardo (2019). Descriptors and indicators of effective teaching practice according to in-service biology teachers. Tecné, Episteme y Didaxis.

[B72-jintelligence-11-00001] Richter Dirk, Kunter Mareike, Klusmann Uta, Lüdtke Oliver, Baumert Jurgen (2011). Professional development across the teaching career: Teachers’ uptake of formal and informal learning opportunities. Teaching and Teacher Education.

[B73-jintelligence-11-00001] Rodríguez Hontanilla Carlos (2015). Smart Organisations as a Response to Overcome Resistance to Organisational Change.

[B74-jintelligence-11-00001] Roig Rosabel, Urrea María Encarnación (2020). Collaborative training in interculturality for Early Childhood and Primary Education teachers. Revista Caribeña de Investigación Educativa.

[B75-jintelligence-11-00001] Rosso Irma, Olivieri Ricardo (2019). The teaching practices of novice teachers at secondary level. Clio and Associates.

[B76-jintelligence-11-00001] Rosso Irma Esther, Alcalá María Teresa (2016). Teacher training and the professional practice of novice teachers in history. Revista del Instituto de Investigaciones en Educació.

[B77-jintelligence-11-00001] Rubio María, Meca Julio Sánchez, Martínez Fulgencio Marín, López Jose Antonio López (2018). Recommendations for the Reporting of Systematic Reviews and Meta-analyses. Annals of Psychology.

[B78-jintelligence-11-00001] Sainz Matilde, Ozaeta Arantzazu (2013). Language teacher training. Characteristics of the activity of an experienced teacher and a novice teacher. Ikastaria. Education Notebooks.

[B79-jintelligence-11-00001] Sánchez Meca Julio, Botella Juan (2010). Systematic reviews and meta-analyses: Tools for professional practice. Papeles del Psicólogo.

[B80-jintelligence-11-00001] Sánchez Povedano Nuria (2015). Multiple intelligences in the classroom. Towards a new model of school and learning. Padres Y Maestros/Journal of Parents and Teachers.

[B81-jintelligence-11-00001] Sánchez-Tarazaga Lucía (2016). The frameworks of teaching competences: Contribution to their study from the European educational policy. Journal of Supranational Policies of Education.

[B82-jintelligence-11-00001] Sangrá Albert, Estévez Iris, Iglesias Verónica, Souto-Seijo Alba (2019). Teacher professional development through learning ecologies: Teacher perspectives. EDUTEC (Electronic Journal of Educational Technology).

[B83-jintelligence-11-00001] Seker Hasan, Deniz Sabahattin (2016). Some Dilemmas Regarding Teacher Training: On the Teacher’s (Not) Being a Role Model. Journal of Education and Training Studies.

[B84-jintelligence-11-00001] Shaaban Eman, Abou Imane (2018). The Impact of Secondary School Teachers’ Training Program on the Professional Development of In-Service Biology Teachers. The Eurasia Proceedings of Educational and Social Sciences.

[B85-jintelligence-11-00001] Sherrington Catherine, Herbert Robert, Maher Christopher, Moseley Anne (2000). PEDro: A database of randomized trials and systematic reviews in physiotherapy. Manual Therapy.

[B86-jintelligence-11-00001] Silva Tim, McKie Allison, Knechtel Virginia, Gleason Philip, Makowsky Libby (2014). Teaching Residency Programs. A Multisite Look at a New Model to Prepare Teachers for High-Need Schools.

[B87-jintelligence-11-00001] Souto Ana Isabel, Rial Antoio Florencio, Talavera Miguel Ángel (2020). Aspects that shape the professional identity of vocational training and guidance teachers. Estudios Sobre Educación.

[B88-jintelligence-11-00001] Suciu Andreia Irina, Mâţă Liliana (2014). Romanian language pre-service teachers’ training for didactic career. Procedia-Social and Behavioral Sciences.

[B89-jintelligence-11-00001] Trust Torrey, Krutka Daniel, Carpenter Jeffrey (2016). Together we are better. Professional learning networks for teachers. Computers and Education.

[B90-jintelligence-11-00001] Umar Irfan Naufal, Yusoff Mohamad Tarmizi Mohd (2014). A study on Malaysian teachers’ level of ICT skills and practices, and its impact on teaching and learning. Procedia-Social and Behavioral Sciences.

[B91-jintelligence-11-00001] UNESCO-ILO (2000). Joint ILO/UNESCO Committee on the Application of the Recommendation Concerning the Status of Teachers. Paper Presented at the Seventh Meeting.

[B92-jintelligence-11-00001] UNESCO-ILO (2009). Joint ILO/UNESCO Committee of Experts on the Application of the Recommendations Concerning Teachers. Paper Presented at the Tenth Meeting.

[B93-jintelligence-11-00001] UNESCO-ILO (2012). Joint ILO/UNESCO Committee of Experts on the Application of the Recommendations Concerning Teachers. Paper Presented at the Eleventh Meeting.

[B94-jintelligence-11-00001] Vallejo Ruiz Mónica, Miguel Nieto Cano Jose, Antonio Portela Pruaño, Begoña Alfageme González María, Ana Torres Soto, Hernández García, Luisa María (2021). Generational diversity among teachers in the workplace: Implications for teacher relationships, identity and development. Journal of Intercultural Management.

[B95-jintelligence-11-00001] Vélaz de Medrano Consuelo, Vaillant Denise (2009). Aprendizaje y Desarrollo Profesional Docente.

[B96-jintelligence-11-00001] Vicente María Inmaculada, Gabari María Inés (2019). Burnout levels in secondary school teachers: A descriptive analytical study. INFAD Journal of Psychology. International Journal of Developmental and Educational Psychology.

[B97-jintelligence-11-00001] Vidiella Judhit, Larrain Verónica (2015). The role of working conditions in the construction of teacher identity. Corporalities, affects and knowledge. Revista Mexicana de Investigación Educativa.

[B98-jintelligence-11-00001] Webber Chris, Hardwell Ashley (2019). ‘Perhaps a Bit Different to What We Did Twenty Years Ago’: Senior Teachers. Perceptions of Outdoor Adventure within Primary Education in England. Sports.

[B99-jintelligence-11-00001] Zapatero Jorge, González María Dolores, Campos Antonio (2018). The initial and in-service training of Physical Education teachers for the application of the competency model. A qualitative study. Revista Complutense de Educación.

[B100-jintelligence-11-00001] Zweig Jacqueline, Stafford Erin, Clements Margaret, Pazzaglia Angela (2015). Professional Experiences of Online Teachers in Wisconsin: Results from a Survey about Training and Challenges.

